# Re: Association Between Stress Hyperglycemia Ratio and Parkinson's Disease Across Different Glucose Metabolism Statuses—A Prospective Study From UK Biobank

**DOI:** 10.1111/ene.70344

**Published:** 2025-09-06

**Authors:** Wenwen Xu, Guoxin Zhang

**Affiliations:** ^1^ Nanjing Qixia District Hospital, Nanjing Nanjing Jiangsu China

Dear Editor,

We read with great interest the recent prospective study by Zhou et al. investigating the association between stress hyperglycemia ratio (SHR) and Parkinson's disease (PD) risk in the UK Biobank cohort [1]. This well‐designed study makes important contributions to our understanding of metabolic factors in neurodegeneration. The authors should be commended for their comprehensive analysis of a large cohort (*n* = 406,271) with robust statistical methodology, sex‐stratified analyses, and thorough covariate adjustment. The identification of sex‐specific associations, particularly the 20% increased PD risk in the highest SHR tertile among men (HR: 1.20, 95% CI: 1.06–1.37), represents a noteworthy finding that advances our knowledge of metabolic risk factors for PD.

However, we would like to raise several methodological considerations that may influence the interpretation of these findings.

## 1 Biological Validity of SHR in Non‐Acute Clinical Settings

The stress hyperglycemia ratio was originally developed by Roberts et al. as a marker of relative hyperglycemia in critically ill hospitalized patients, where it captures the acute glycemic response to physiological stress [2]. The formula [FPG (mmol/L)]/[1.59 × HbA1c (%) − 2.59] was specifically designed to quantify the discrepancy between admission glucose levels and chronic glycemic status in acutely stressed patients. Recent studies have consistently validated SHR as a predictor of adverse outcomes in acute cardiovascular and critical care settings [3]. In the current study, however, SHR was calculated using baseline fasting glucose measurements from healthy community‐dwelling participants during routine health assessments.

This fundamental difference raises important questions about the biological interpretation of SHR in non‐acute settings. Unlike the original context where elevated SHR reflects counter‐regulatory hormone release and acute stress responses, elevated baseline SHR in healthy individuals may simply represent chronic glucose dysregulation or normal physiological variation. The term “stress hyperglycemia ratio” becomes potentially misleading when applied to baseline measurements in asymptomatic populations, as it implies an acute stress response that may not be physiologically present.

## 2 Methodological Limitations and Their Impact on Result Interpretation

Several methodological considerations warrant discussion. First, the reliance on single‐point glucose and HbA1c measurements at baseline may inadequately capture long‐term glycemic patterns. Given PD's lengthy prodromal phase, which can span years to decades [4], a single SHR calculation may not reflect the cumulative metabolic stress burden relevant to neurodegeneration. Additionally, glycemic variability—an important aspect of metabolic dysfunction—cannot be assessed through baseline measurements alone.

Second, while the authors excluded participants diagnosed with PD within the first 2 years of follow‐up, this approach may be insufficient to address reverse causation bias. Recent research has demonstrated that autonomic dysfunction, including impaired glucose homeostasis, can manifest during PD's pre‐motor phase and may precede clinical diagnosis by many years [5]. The observed association between SHR and PD risk could, therefore, reflect early subclinical neurodegeneration rather than representing a true causal risk factor.

Third, the study's extensive subgroup analyses—stratified by sex and three diabetes categories—increase the likelihood of type I error through multiple testing. While the authors report nominally significant findings in specific subgroups, formal statistical interaction testing and appropriate correction for multiple comparisons would strengthen the robustness of these conclusions.

## 3 Clinical Implications and Future Directions

Despite these limitations, the study's identification of sex‐specific metabolic risk patterns for PD provides valuable insights worthy of further investigation. We suggest that future research should: (1) employ repeated metabolic measurements to better characterize long‐term glycemic patterns; (2) investigate the biological mechanisms underlying observed sex differences in metabolic susceptibility to neurodegeneration; (3) explore whether metabolic interventions can modify PD risk in high‐risk populations; (4) develop more appropriate metrics for assessing chronic metabolic stress in community‐based cohorts.

The authors' work represents an important contribution toward understanding the complex interplay between metabolism and neurodegeneration. However, careful consideration of the biological validity of SHR in non‐acute settings and acknowledgment of the methodological limitations discussed above will be essential for appropriately translating these findings into clinical practice and future research directions.

## Author Contributions

**Wenwen Xu:** writing – original draft. **Guoxin Zhang:** writing – original draft, writing – review and editing.

## Ethics Statement

The authors have nothing to report.

## Conflicts of Interest

The authors declare no conflicts of interest.

## Data Availability

The data that support the findings of this study are available from the corresponding author upon reasonable request.
